# Cortical Hierarchies Perform Bayesian Causal Inference in Multisensory Perception

**DOI:** 10.1371/journal.pbio.1002073

**Published:** 2015-02-24

**Authors:** Tim Rohe, Uta Noppeney

**Affiliations:** 1 Max Planck Institute for Biological Cybernetics, Tuebingen, Germany; 2 Computational Neuroscience and Cognitive Robotics Centre, University of Birmingham, Birmingham, United Kingdom; Glasgow University, UNITED KINGDOM

## Abstract

To form a veridical percept of the environment, the brain needs to integrate sensory signals from a common source but segregate those from independent sources. Thus, perception inherently relies on solving the “causal inference problem.” Behaviorally, humans solve this problem optimally as predicted by Bayesian Causal Inference; yet, the underlying neural mechanisms are unexplored. Combining psychophysics, Bayesian modeling, functional magnetic resonance imaging (fMRI), and multivariate decoding in an audiovisual spatial localization task, we demonstrate that Bayesian Causal Inference is performed by a hierarchy of multisensory processes in the human brain. At the bottom of the hierarchy, in auditory and visual areas, location is represented on the basis that the two signals are generated by independent sources (= segregation). At the next stage, in posterior intraparietal sulcus, location is estimated under the assumption that the two signals are from a common source (= forced fusion). Only at the top of the hierarchy, in anterior intraparietal sulcus, the uncertainty about the causal structure of the world is taken into account and sensory signals are combined as predicted by Bayesian Causal Inference. Characterizing the computational operations of signal interactions reveals the hierarchical nature of multisensory perception in human neocortex. It unravels how the brain accomplishes Bayesian Causal Inference, a statistical computation fundamental for perception and cognition. Our results demonstrate how the brain combines information in the face of uncertainty about the underlying causal structure of the world.

## Abstract 2

The human brain uses Bayesian Causal Inference to integrate and segregate information by encoding multiple estimates of spatial relationships at different levels of the auditory and visual processing hierarchies. Read the accompanying Primer.

## Author Summary

How can the brain integrate signals into a veridical percept of the environment without knowing whether they pertain to same or different events? For example, I can hear a bird and I can see a bird, but is it one bird singing on the branch, or is it two birds (one sitting on the branch and the other singing in the bush)? Recent studies demonstrate that human observers solve this problem optimally as predicted by Bayesian Causal Inference; yet, the neural mechanisms remain unclear. By combining psychophysics, Bayesian modelling, functional magnetic resonance imaging (fMRI), and multivariate decoding in an audiovisual localization task, we show that Bayesian Causal Inference is performed by a neural hierarchy of multisensory processes. At the bottom of the hierarchy, in auditory and visual areas, location is represented on the basis that the two signals are generated by independent sources (= segregation). At the next stage, in posterior intraparietal sulcus, location is estimated under the assumption that the two signals are from a common source (= forced fusion). Only at the top of the hierarchy, in anterior intraparietal sulcus, the uncertainty about the world’s causal structure is taken into account and sensory signals are combined as predicted by Bayesian Causal Inference.

## Introduction

Our senses are constantly bombarded with many different signals. Imagine you are crossing a street and suddenly hear a loud motor noise. Is that motor noise coming from the car on the opposite side of the street or from a rapidly approaching car that you have not yet spotted? To locate the source of the motor noise more precisely, you should integrate the auditory signal with the sight of the car only if the two inputs pertain to the same object. Thus, estimating an environmental property (e.g., spatial location) in multisensory perception inherently relies on inferring whether sensory signals are caused by common or independent sources [1,2].

Past research in perception and cue combination has mostly ignored the causal inference problem and focused on the special case in which sensory signals arise from a common source. A large body of research has demonstrated that observers integrate signals near-optimally weighted by their reliability in these “forced fusion” settings [3–9]. Yet, in our complex natural environment forced fusion would be detrimental and the brain needs to balance integration and segregation according to the underlying causal structure (i.e., common versus independent sources) [10].

Hierarchical Bayesian Causal Inference provides a rational strategy to arbitrate between information integration and segregation in perception and cognition. In case of a common source, signals should be integrated weighted by their relative sensory reliabilities [3,4]. In case of independent sources, they should be processed independently. Critically, the observer does not know the underlying causal structure and needs to infer it from spatiotemporal or higher order (e.g., semantic) congruency cues [2]. To account for the uncertainty about the causal structure, an observer should compute a final estimate by averaging the estimates (e.g., spatial location) under the two potential causal structures weighted by the posterior probabilities of these structures (i.e., model averaging).

Indeed, recent psychophysics and modeling efforts have demonstrated that human observers locate audiovisual signal sources in line with Bayesian Causal Inference by combining the spatial estimates under the assumptions of common and independent sources weighted by their posterior probabilities [2]. For small spatial disparities, audiovisual spatial signals are integrated weighted by their relative sensory reliabilities leading to strong crossmodal spatial biases [3]; for large spatial disparities, these crossmodal biases are greatly attenuated [11,12], because the final spatial estimate relies predominantly on the segregated option.

However, the neural mechanisms that enable Bayesian Causal Inference are unknown. In particular, it is unclear whether the brain encodes the spatial estimates under the assumptions of common and independent sources in order to perform Bayesian Causal Inference. Does the brain explicitly represent several spatial estimates that enter into Bayesian Causal Inference?

## Results and Discussion

**Fig 1:**
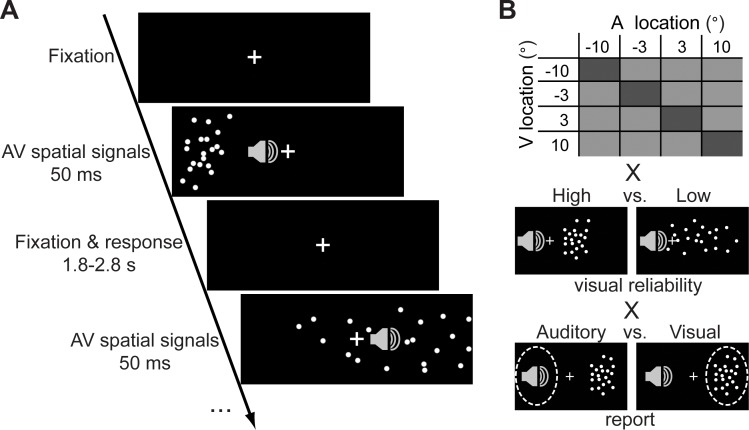
Example trial and experimental design. (A) In a spatial ventriloquist paradigm, participants were presented with synchronous audiovisual (AV) signals originating from four possible locations along the azimuth. The visual signal was a cloud of white dots. The auditory signal was a brief burst of white noise. Participants localized either the auditory or the visual signal (n.b. for illustrational purposes the visual angles of the cloud have been scaled in a non-uniform fashion in this scheme). (B) The four-factorial experimental design manipulated (1) the location of the visual (V) signal (−10°, −3.3°, 3.3°, 10°) (2) the location of the auditory (A) signal (−10°, −3.3°, 3.3°, 10°), (3) the reliability of the visual signal (high versus low standard deviation of the visual cloud), and (4) task-relevance (auditory versus visual report).

We combined psychophysics, Bayesian statistical modeling, and a multivariate functional magnetic resonance imaging (fMRI) decoding approach to characterize how the human brain performs Bayesian Causal Inference along the auditory [13] and visual [14] spatial cortical hierarchies. During fMRI scanning, we presented five participants with synchronous auditory (white noise) and visual (Gaussian cloud of dots) spatial signals that were independently sampled from four possible locations along the azimuth (i.e., −10°, −3.3°, 3.3°, or 10°) (Fig 1A). Further, we manipulated the reliability of the visual signal by varying the standard deviation of the visual cloud (2° or 14° standard deviation). Participants were asked selectively to report either the visual or the auditory signal location (without feed-back). Thus, the 4 (auditory locations) × 4 (visual locations) × 2 (visual reliability) × 2 (visual versus auditory report) factorial design included 64 conditions (Fig 1B). Importantly, as auditory and visual spatial locations were sampled independently on each trial, our design implicitly manipulated audiovisual spatial disparity, a critical cue informing the brain whether signals emanate from common or independent sources (cf. supporting S5 Table).

### Behavioral Results

**Fig 2:**
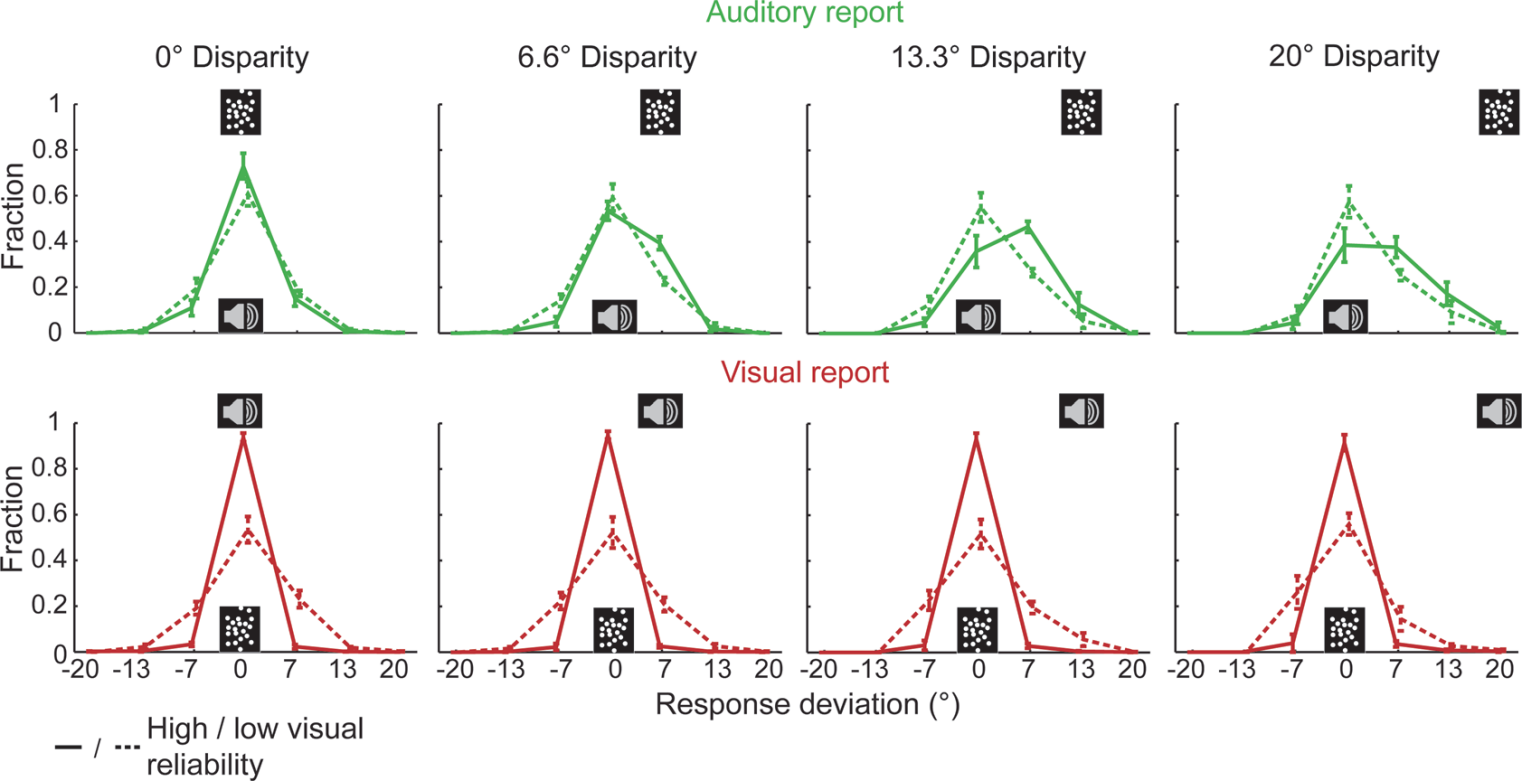
Histograms of response deviations (across-subjects mean fraction ± standard error of the mean [SEM]) as a function of (i) task relevance (i.e., auditory versus visual report) (ii) audiovisual disparity, and (iii) visual reliability. If participants were able to locate the task-relevant auditory or visual signal precisely, the histogram over response deviations would reduce to a delta function centered on zero. The histograms of response deviations for auditory report indicate that a spatially disparate visual signal biases participants’ perceived sound location in particular when the visual signal is reliable. In each panel, stimulus symbols (i.e., auditory: loudspeaker; visual: cloud of dots) indicate the location of the task-relevant signal (centered on zero) and the task-irrelevant signal (centered on the discrepant spatial location). The data used to make this figure are available in file S1 Data.

At the behavioral level, we first investigated how participants integrate and segregate sensory signals for auditory and visual spatial localization. Fig 2 shows the histograms of response deviations as a function of task-relevance (i.e., auditory versus visual report), audiovisual spatial disparity, and visual reliability. If participants were able to determine the location of the task-relevant auditory or visual signal precisely, the histogram over response deviations would reduce to a delta function centered on zero. Thus, the difference in widths of the histograms for auditory and visual report indicates that participants were less precise when locating auditory (green) as compared to the visual signals (red). Likewise, as expected visual localization was less precise for low (red dashed) relative to high visual (red solid) reliability. Importantly, for auditory localization, the response distribution was shifted towards a concurrent spatially discrepant visual signal. This visual spatial bias on the perceived auditory location was increased when the visual signal was reliable, thus replicating the classical profile of the spatial ventriloquist effect [3]. Moreover, it was more pronounced for 13.3° than for 20° disparity. In other words, as expected under Bayesian Causal Inference, the influence of a concurrent visual signal on the perceived auditory location was attenuated for large spatial discrepancies, when it was less likely that auditory and visual signals came from a common source.

Next, we analyzed visual and auditory localization reports more formally by comparing three models. (i) The full-segregation model assumes that auditory and visual signals are processed independently. (ii) The forced-fusion model assumes that auditory and visual signals are integrated weighted by their reliabilities in a mandatory fashion irrespective of the environmental causal structure. (iii) The Bayesian Causal Inference model computes a final auditory (or visual) spatial estimate by averaging the spatial estimates under forced-fusion and full-segregation assumptions weighted by the posterior probabilities of each causal structure (i.e., model averaging, see S3 and S4 Tables for other decision functions). Using a maximum likelihood procedure, we fitted the parameters (e.g., visual variances σ_V1_^2^ − σ_V2_^2^ for the two reliability levels) of the three models individually to each participant’s behavioral localization responses. Bayesian model comparison corroborated previous results [2] and demonstrated that the Bayesian Causal Inference model outperformed the full-segregation and forced-fusion models (82.4% variance explained, exceedance probability of 0.95) (Table 1). In other words, human observers integrate audiovisual spatial signals predominantly when they are close in space and hence likely to come from a common source.

**Table 1:** Model parameters (across-subjects mean ± standard error of the mean) and fit indices of the three computational models.

**Model**	**p_C_**	**σ_P_**	**σ_A_**	**σ_V1_**	**σ_V2_**	**R^2^**	**relBIC_Group_**	**EP**
Causal Inference (model averaging)	0.48 ± 0.10	14.9 ± 3.8	17.1 ± 7.0	3.8 ± 0.5	8.3 ± 0.8	82.4 ± 3.8	0	0.953
Forced fusion	—	14.4 ± 1.6	14.3 ± 2.0	6.5 ± 0.5	10.7 ± 0.7	60.7 ± 3.4	7,027.5	0.017
Full segregation	—	13.1 ± 2.7	24.1 ± 9.9	4.1 ± 0.7	7.5 ± 0.9	79.1 ± 4.3	992.6	0.030

p_C_, prior common-source probability; σ_P_, standard deviation of the spatial prior (in °); σ_A_, standard deviation of the auditory likelihood (in °); σ_V_, standard deviation of the visual likelihood at two levels of visual reliability (1, high; 2, low) (in °); R^2^, coefficient of determination; relBIC_Group_, Bayesian information criterion at the group level, i.e., subject-specific BICs summed over all subjects (BIC = LL − 0.5 M ln(N), LL = log likelihood, M = number of parameters, N = number of data points) of a model relative to the Bayesian Causal Inference (“model averaging”) model (n.b. a smaller relBIC_Group_ indicates that a model provides a better explanation of our data); EP, exceedance probability, i.e., probability that a model is more likely than any other model.

### fMRI Results

**Fig 3:**
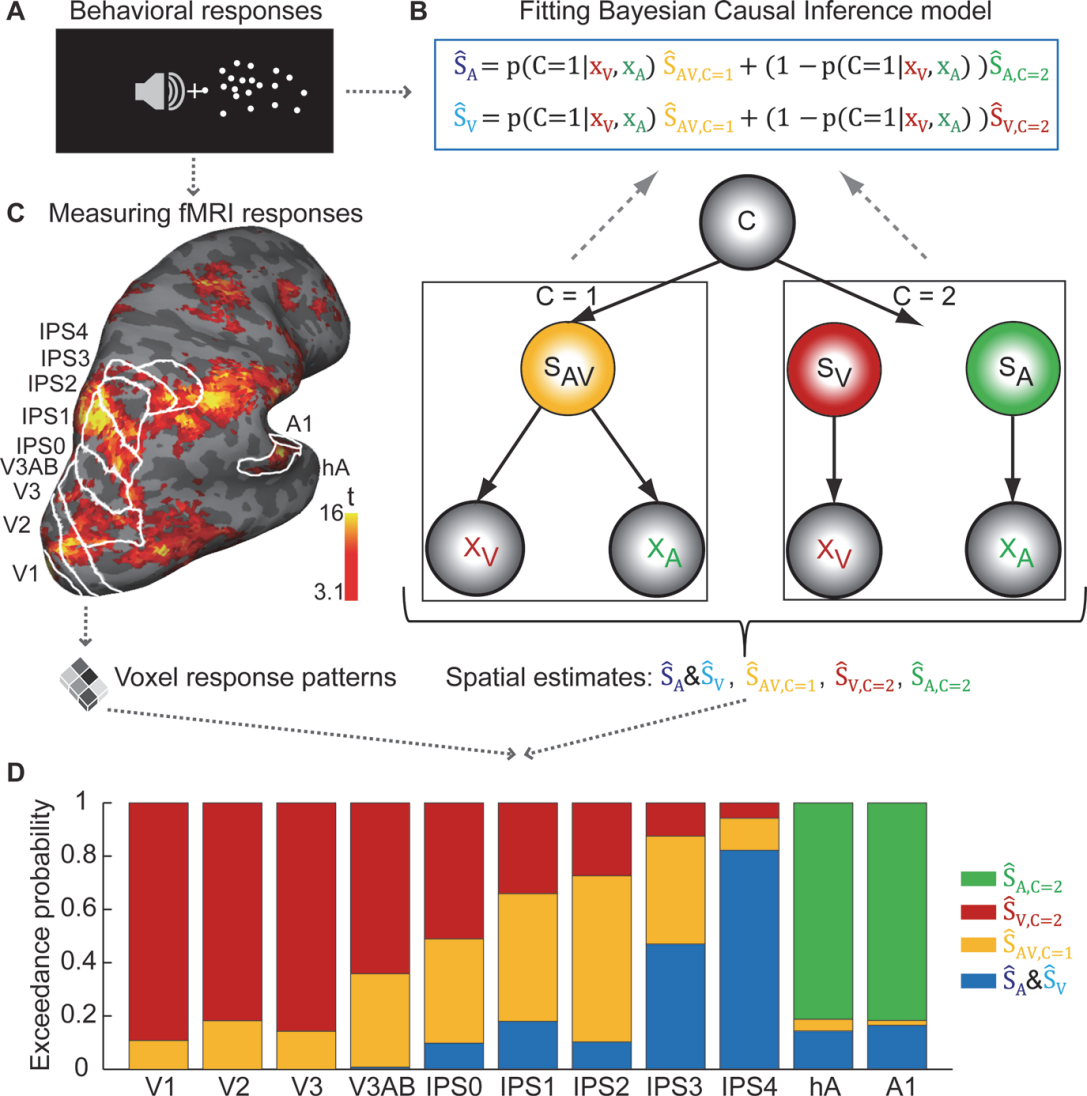
Bayesian Causal Inference model and cortical hierarchies. (A) Participants were presented with auditory and visual spatial signals. We recorded participants’ psychophysical localization responses and fMRI BOLD responses. (B) The Bayesian Causal Inference model [2] was fitted to participants’ localization responses and then used to obtain four spatial estimates for each condition: the unisensory auditory (Ŝ_A,C=2_) and visual (Ŝ_V,C=2_) estimates under full segregation (C = 2), the forced-fusion estimate (Ŝ_AV,C=1_) under full integration (C = 1), and the final spatial estimate (Ŝ_A_, Ŝ_V_) that averages the task-relevant unisensory and the forced-fusion estimate weighted by the posterior probability of each causal structure (i.e., for a common source: p(C = 1|x_A_, x_V_) or independent sources: 1 − p(C = 1|x_A_, x_V_). (C) fMRI voxel response patterns were obtained from regions along the visual and auditory hierarchies (V, visual sensory regions; A1, primary auditory cortex; hA, higher auditory area; IPS, intraparietal sulcus). (D) Exceedance probabilities index the belief that a given spatial estimate is more likely represented within a region of interest than any other spatial estimate. The exceedance probabilities for the different spatial estimates are indexed in the length of the colored areas of each bar (n.b. the *y*-axis indicates the cumulative exceedance probabilities). The data used to make this figure are available in file S1 Data.

Next, we asked how Bayesian Causal Inference emerged along the auditory and visual cortical hierarchies (Fig 3). In particular, Bayesian Causal Inference entails four spatial estimates: the full-segregation unisensory (i) auditory (Ŝ_A,C = 2_) and (ii) visual estimates (Ŝ_V,C = 2_), (iii) the “audiovisual forced-fusion estimate” (Ŝ_AV,C = 1_), and (iv) the final Bayesian Causal Inference estimate (Ŝ_A_ & Ŝ_V_, pooled over conditions of auditory and visual report) that is obtained by averaging the forced-fusion and the task-relevant unisensory estimates weighted by the posterior probability of each causal structure. We obtained these four spatial estimates for each of the 64 conditions and each participant from the Causal Inference model fitted individually to participant’s behavioral data (Fig 3B, bottom). Using cross-validation, we trained a support vector regression model to decode each of these four spatial estimates from fMRI voxel response patterns in regions along the cortical hierarchies defined by visual retinotopic and auditory localizers (Fig 3C). We quantified the decoding accuracies for each of these four spatial estimates in terms of their correlation between (i) the spatial estimates obtained from the Causal Inference model fitted individually to participants’ localization responses (i.e., training labels for fMRI decoding) and (ii) the spatial estimates decoded from fMRI voxel response patterns. To determine which of the four spatial estimates is primarily encoded in a particular region, we computed the exceedance probability that a correlation coefficient of one spatial estimate was greater than that of any other spatial estimate by bootstrapping the decoding accuracies (Fig 3D).

The profile of exceedance probabilities demonstrates that Bayesian Causal Inference is performed by a hierarchy of multisensory processes in the human brain: At the bottom of the hierarchy, in auditory and visual areas, location is represented on the basis that the two signals are generated by independent sources. Thus, primary sensory areas predominantly encoded the spatial estimate of their preferred sensory modality under information segregation, even though they also showed limited multisensory influences as previously reported [15–21]. At the next stage, in posterior intraparietal sulcus (IPS1–2), location is estimated under the assumption that the two signals are from a common source. In other words, IPS1–2 represented primarily the reliability-weighted integration estimate under forced-fusion assumptions. It is only at the top of the hierarchy, in anterior intraparietal sulcus (IPS3–4), that the uncertainty about whether signals are generated by common or independent sources is taken into account. As predicted by Bayesian Causal Inference, location is estimated in IPS3–4 by combining the full-segregation and the forced-fusion estimates weighted by the posterior probabilities of common and independent sources. Thus, according to Bayesian Causal Inference the spatial estimates in IPS3–4 should be influenced by task-irrelevant sensory signals primarily for small spatial disparities, when signals were likely to be generated by a common event. Critically, while no region could uniquely be assigned one type of spatial estimate, the profile of exceedance probabilities reveals a hierarchical organization of the computational operations in human neocortex.

Recent elegant neurophysiological research in non-human primates has shown how single neurons and neuronal populations implement reliability-weighted integration under forced-fusion assumptions [22–24]. In other words, they presented visual and vestibular signals only with a very small discrepancy, so that signals could be assumed to arise from a common source. Yet, to our knowledge this is the first neuroimaging study that moves beyond traditional forced-fusion models and demonstrates how the brain performs hierarchical Bayesian Causal Inference [2]. Thus, future neurophysiological and modelling research will need to define how single neurons and neuronal populations implement computational operations of Bayesian Causal Inference, potentially via probabilistic population codes [25].

Accumulating evidence has suggested that multisensory interactions are pervasive in human neocortex [18,26–31] starting already at the primary cortical level [15–21]. Indeed, our multivariate decoding analysis also revealed multisensory influences ubiquitously along the auditory and visual processing streams with limited multisensory influences emerging already in primary sensory areas.

To link our study more closely with previous fMRI results of spatial ventriloquism, we have interrogated our data also with a conventional univariate analysis of regional blood-oxygen-level dependent (BOLD) responses. Converging with our model-based findings, this conventional analysis also suggested that low-level sensory areas are predominantly driven by signals of their preferred sensory modality (e.g., visual cortex by visual signals). Yet, in line with previous reports [27,31], visual signals influenced the BOLD response already in the “higher auditory area” (hA) encompassing the planum temporale. Moreover, while activations in parietal areas were still influenced by visual location, they were progressively susceptible to effects of task-context mediated either directly or in interaction with visual reliability (see supporting results and discussion in S1 Fig, S2 Table, and S1 Text). Thus, both the regional BOLD response and the spatial representations encoded in parietal areas and to some extent in auditory areas were influenced by whether the location of the visual or the auditory signal needed to be attended to and reported in line with the principles of Bayesian Causal Inference.

As the current paradigm manipulated the factor of task-relevance over sessions, participants knew the sensory modality that needed to be reported prior to stimulus presentation. Thus, the regional BOLD-response in higher auditory cortices is likely to be modulated by attentional top-down effects [32–36]. Future studies may investigate Bayesian Causal Inference when auditory and visual report trials are presented in a randomized fashion to minimize attention- and expectation-related effects. Alternatively, studies could factorially manipulate (i) the attended and (ii) the reported sensory modality. For instance, participants may be cued to attend to the auditory modality prior to stimulus presentation and yet be instructed to report the visual modality after stimulus presentation.

Yet, despite these attempts Bayesian Causal Inference may inherently entail processes associated with “attentional modulation” in a wider sense, as it computationally requires combining the multisensory forced-fusion estimate with the “task-relevant” unisensory estimate. Critically, however, the effects of attentional modulation or task-relevance invoked by Bayesian Causal Inference should interact with the spatial discrepancy between the sensory signals. Effects of task-relevance should be most pronounced for large spatial discrepancies.

In conclusion, the multivariate analysis based on Bayesian Causal Inference moves significantly beyond identifying multisensory interactions, towards characterizing their computational operations that prove to differ across cortical levels. This methodological approach provides a novel hierarchical perspective on multisensory integration in human neocortex. We demonstrate that the brain simultaneously encodes multiple spatial estimates based on segregation, forced fusion, and model averaging along the cortical hierarchy. Only at the top of the hierarchy, higher-order anterior IPS3–4 takes into account the uncertainty about the causal structure of the world and combines sensory signals as predicted by Bayesian Causal Inference. To our knowledge, this study is the first compelling demonstration of how the brain performs Bayesian Causal Inference, a statistical operation fundamental for perception and cognition.

## Materials and Methods

### Participants

The study was approved by the human research review committee of the University of Tuebingen (approval number 432 2007 BO1). After giving written informed consent, six healthy volunteers without a history of neurological or psychiatric disorders (all university students or graduates; 2 female; mean age 28.8 years, range 22–36 years) participated in the fMRI study. All participants had normal or corrected-to-normal vision and reported normal hearing. One participant was excluded because of excessive head motion (4.21/3.52 standard deviations above the mean of the translational/rotational volume-wise head motion based on the included five participants).

### Stimuli

The visual stimulus was a cloud of 20 white dots (diameter: 0.43° visual angle) sampled from a bivariate Gaussian with a vertical standard deviation of 2.5° and a horizontal standard deviation of 2° or 14° presented on a black background (i.e., 100% contrast). Participants were told that the 20 dots were generated by one underlying source in the center of the cloud.

The auditory stimulus was a burst of white noise with a 5 ms on/off ramp. To create a virtual auditory spatial signal, the noise was convolved with spatially specific head-related transfer functions (HRTFs) thereby providing binaural (interaural time and amplitude differences) and monoaural spatial filtering signals. The HRTFs were pseudo-individualized by matching participants’ head width, height, depth, and circumference to the anthropometry of participants in the CIPIC database [37]. HRTFs from the available locations in the database were interpolated to the desired location of the auditory signal. The behavioral responses from the auditory localizer session (see below) indicated that participants were able to localize the virtual auditory spatial signals in the magnetic resonance (MR) scanner. They were significantly better than chance at discriminating whether two subsequent auditory signals were presented from the same or different locations (mean accuracy = 0.88; mean d’ = 3.14, *p* = 0.001 in a one sample *t*-test against zero).

### Experimental Design

In a spatial ventriloquist paradigm, participants were presented with synchronous, yet spatially congruent or disparate visual and auditory signals (Fig 1A). On each trial, visual and auditory locations were independently sampled from four possible locations along the azimuth (i.e., −10°, −3.3°, 3.3°, or 10°) leading to four levels of spatial discrepancy (i.e., 0°, 6.6°, 13.3°, or 20°). In addition, we manipulated the reliability of the visual signal by setting the horizontal standard deviation of the Gaussian cloud to 2° (high reliability) or 14° (low reliability) visual angle. In an inter-sensory selective-attention paradigm, participants reported their auditory or visual perceived signal location and ignored signals in the other modality. For the visual modality, they were asked to determine the location of the center of the visual cloud of dots. Hence, the 4 × 4 × 2 × 2 factorial design manipulated (i) the location of the visual stimulus ({−10°, −3.3°, 3.3°, 10°}, i.e., the mean of the Gaussian); (ii) the location of the auditory stimulus ({−10°, −3.3°, 3.3°, 10°}); (iii) the reliability of the visual signal ({2°,14°}, standard deviation of the Gaussian); and (iv) task-relevance (auditory-/visual-selective report) resulting in 64 conditions (Fig 1B). Please note that in contrast to our inter-sensory attention paradigm, Koerding and colleagues [2] employed a dual task paradigm where participants reported auditory and visual locations on each trial. Thus, the two paradigms differ in terms of attentional and task-induced processes.

On each trial, synchronous audiovisual spatial signals were presented for 50 ms followed by a variable inter-stimulus fixation interval from 1.75–2.75 s. Participants localized the signal in the task-relevant sensory modality as accurately as possible by pushing one of four spatially corresponding buttons. Throughout the experiment, they fixated a central cross (1.6° diameter).

To maximize design efficiency, stimuli and conditions were presented in a pseudorandomized fashion. Only the factor task-relevance was held constant within a session and counterbalanced across sessions. In each session, each of the 32 audiovisual spatial stimuli was presented exactly 11 times either under auditory- or visual-selective report. On average, 5.9% of the trials were interspersed as null-events in the sequence of 352 stimuli per session. Each participant completed 20 sessions (ten auditory and ten visual localization reports; apart from one participant who performed nine auditory and 11 visual localization sessions). Before the fMRI study, participants completed one practice session outside the scanner.

### Experimental Setup

Audiovisual stimuli were presented using Psychtoolbox 3.09 (www.psychtoolbox.org) [38] running under MATLAB R2010a (MathWorks). Auditory stimuli were presented at ~75 dB SPL using MR-compatible headphones (MR Confon). Visual stimuli were back-projected onto a Plexiglas screen using an LCoS projector (JVC DLA-SX21). Participants viewed the screen through an extra-wide mirror mounted on the MR head-coil resulting in a horizontal visual field of approximately 76° at a viewing distance of 26 cm. Participants performed the localization task using an MR-compatible custom-built button device. Participants’ eye movements and fixation were monitored by recording participants’ pupil location using an MR-compatible custom-build infrared camera (sampling rate 50 Hz) mounted in front of the participants’ right eye and iView software 2.2.4 (SensoMotoric Instruments).

### Eye Movement Recording and Analysis

To address potential concerns that our results may be confounded by eye movements, we evaluated participants’ eye movements based on eye tracking data recorded concurrently during fMRI acquisition. Eye recordings were calibrated with standard eccentricities between ±3° and ±10° to determine the deviation from the fixation cross. Fixation position was post-hoc offset corrected. Eye position data were automatically corrected for blinks and converted to radial velocity. For each condition, the number of saccades (defined by a radial eye-velocity threshold of 15° s^−1^ for a minimum of 60 ms duration and radial amplitude larger than 1°) were quantified (0–875 ms after stimulus onset). Fixation was well maintained throughout the experiment with post-stimulus saccades detected in only 2.293% ± 1.043% (mean ± SEM) of the trials. Moreover, 4 (visual location) × 4 (auditory location) × 2 (visual reliability) × 2 (visual versus auditory report) repeated measure ANOVAs performed separately for (i) % saccades or (ii) % eye blinks revealed no significant main effects or interactions.

### Behavioral Analysis

To characterize how participants integrate auditory and visual signals into spatial representations, we computed the deviation between the responded location and the mean responded location in the corresponding congruent condition for each trial and in each subject. For instance, for trial i (e.g., auditory location = 3.3°, visual location = −3.3°, visual reliability = low, visual report) we computed the response deviation by comparing the responded visual location in trial i to the mean responded visual location for the corresponding congruent condition (e.g., auditory location = −3.3°, visual location = −3.3°, visual reliability = low, visual report). We then averaged the individual histograms of response deviations across subjects (Fig 2, for an additional analysis of response accuracy see supporting results in S1 Table and S1 Text). Fig 2 shows the histograms of the response deviations as a function of task-relevance, visual reliability and audiovisual disparity (i.e., disparity = visual location − auditory location). Please note that we flipped the histograms for negative spatial disparities and auditory report and the histograms for positive spatial disparities and visual report, so that for both types of reports increasing disparity corresponded to a rightward shift of the task-irrelevant signal in Fig 2. We then combined the histograms for positive and negative spatial disparities to reduce the number of conditions and the complexity of Fig 2.

### Bayesian Causal Inference Model

Details of the Bayesian Causal Inference model of audiovisual perception can be found in Koerding and colleagues [2]. The generative model (Fig 3B) assumes that common (C = 1) or independent (C = 2) sources are determined by sampling from a binomial distribution with the common-source prior P(C = 1) = p_common_. For a common source, the “true” location S_AV_ is drawn from the spatial prior distribution N(μ_P_, σ_P_). For two independent causes, the “true” auditory (S_A_) and visual (S_V_) locations are drawn independently from this spatial prior distribution. For the spatial prior distribution, we assumed a central bias (i.e., μ_P_ = 0). We introduced sensory noise by drawing x_A_ and x_V_ independently from normal distributions centered on the true auditory (respectively, visual) locations with parameters σ_A_ (respectively, σ_V_). Thus, the generative model included the following free parameters: the common-source prior p_common_, the spatial prior variance σ_P_^2^, the auditory variance σ_A_^2^, and the two visual variances σ_V_^2^ corresponding to the two visual reliability levels.

Under the assumption of a squared loss function, the posterior probability of the underlying causal structure can be inferred by combining the common-source prior with the sensory evidence according to Bayes rule (cf. S5 Table):

$$\text{p}\left(\text{C}\;=\;1|{\text{x}}_{\text{A}},{\text{x}}_{\text{V}}\right)\;=\;\frac{\text{p}\left({\text{x}}_{\text{A}},{\text{x}}_{\text{V}}|\text{C}=1\right){\text{p}}_{\text{common}}}{\text{p}\left({\text{x}}_{\text{A}},{\text{x}}_{\text{V}}\right)}$$ (1)

In the case of a common source (C = 1) (Fig 3B left), the optimal estimate of the audiovisual location is a reliability-weighted average of the auditory and visual percepts and the spatial prior.

$${\hat{\text{S}}}_{\text{AV},\text{C}=1}\;=\;\frac{\frac{{\text{x}}_{\text{A}}}{{\sigma_{\text{A}}}^{2}}+\frac{{\text{x}}_{\text{V}}}{{\sigma_{\text{V}}}^{2}}+\frac{\mu_{\text{P}}}{{\sigma_{\text{P}}}^{2}}}{\frac{1}{{\sigma_{\text{A}}}^{2}}+\frac{1}{{\sigma_{\text{V}}}^{2}}+\frac{1}{{\sigma_{\text{P}}}^{2}}}$$ (2)

In the case of independent sources (C = 2) (Fig 3B right), the optimal estimates of the auditory and visual signal locations (for the auditory and visual location report, respectively) are independent from each other.

$${\hat{\text{S}}}_{\text{A},\text{C}=2}\;=\;\frac{\frac{{\text{x}}_{\text{A}}}{{\sigma_{\text{A}}}^{2}}+\frac{\mu_{\text{P}}}{{\sigma_{\text{P}}}^{2}}}{\frac{1}{{\sigma_{\text{A}}}^{2}}+\frac{1}{{\sigma_{\text{P}}}^{2}}},\;{\hat{\text{S}}}_{\text{V},\text{C}=2}\;=\;\frac{\frac{{\text{x}}_{\text{V}}}{{\sigma_{\text{V}}}^{2}}+\frac{\mu_{\text{P}}}{{\sigma_{\text{P}}}^{2}}}{\frac{1}{{\sigma_{\text{V}}}^{2}}+\frac{1}{{\sigma_{\text{P}}}^{2}}}$$ (3)

To provide a final estimate of the auditory and visual locations, the brain can combine the estimates under the two causal structures using various decision functions such as “model averaging,” “model selection,” and “probability matching” [39]. In the main paper, we present results using “model averaging” as the decision function that was associated with the highest model evidence and exceedance probability at the group level (see S4 Table; please note that at the within-subject level, model averaging was the most likely decision strategy in only three subjects, see S3 Table, and Wozny and colleagues [39]). According to the “model averaging” strategy, the brain combines the integrated forced-fusion spatial estimate with the segregated, task-relevant unisensory (i.e., either auditory or visual) spatial estimates weighted in proportion to the posterior probability of the underlying causal structures.

$${\hat{\text{S}}}_{\text{A}}=\text{p}(\text{C}=1|{\text{x}}_{\text{A}},{\text{x}}_{\text{V}})\;{\hat{\text{S}}}_{\text{AV},\text{C}=1}+(1-\text{p}(\text{C}=1|{\text{x}}_{\text{A}},{\text{x}}_{\text{V}}))\;{\hat{\text{S}}}_{\text{A},\text{C}=2}$$ (4)

$${\hat{\text{S}}}_{\text{V}}=\text{p}(\text{C}=1|{\text{x}}_{\text{A}},{\text{x}}_{\text{V}})\;{\hat{\text{S}}}_{\text{AV},\text{C}=1}+(1-\text{p}(\text{C}=1|{\text{x}}_{\text{A}},{\text{x}}_{\text{V}}))\;{\hat{\text{S}}}_{\text{V},\text{C}=2}$$ (5)

Thus, Bayesian Causal Inference formally requires three spatial estimates (Ŝ_AV,C = 1_, Ŝ_A,C = 2_, Ŝ_V,C = 2_) which are combined weighted by the posterior probability of each causal structure into a final estimate (Ŝ_A_ / Ŝ_V_, depending on which sensory modality is task-relevant).

We evaluated whether and how participants integrate auditory and visual signals based on their behavioral localization responses by comparing three models: (i) The observers may process and report auditory and visual signals independently (i.e., the full-segregation model, Equation 3). (ii) They may integrate auditory and visual signals in a mandatory fashion irrespective of spatial disparity (i.e., the forced-fusion model, Equation 2). (iii) The observer may perform Bayesian Causal Inference, i.e., combine estimates from the forced-fusion and the task-relevant estimate from the full-segregation model weighted by the probability of the underlying causal structures (Equations 4 and 5, i.e., model averaging, for other decision functions see S3 Table and S4 Table).

To arbitrate between full segregation, forced fusion, and Bayesian Causal Inference, we fitted each model to participants’ localization responses (Table 1) based on the predicted distributions of the auditory spatial estimates (i.e., p(Ŝ_A_|S_A_,S_V_)) and the visual spatial estimates (i.e., p(Ŝ_V_|S_A_,S_V_)). These distributions were obtained by marginalizing over the internal variables x_A_ and x_V_ that are not accessible to the experimenter (for further details of the fitting procedure see Koerding and colleagues [2]). These distributions were generated by simulating x_A_ and x_V_ 5,000 times for each of the 64 conditions and inferring Ŝ_A_ and Ŝ_V_ from Equations 1–5. To link p(Ŝ_A_|S_A_,S_V_) and p(Ŝ_V_|S_A_,S_V_) to participants’ auditory or visual discrete localization responses, we assumed that participants selected the button that is closest to Ŝ_A_ or Ŝ_V_ and binned the Ŝ_A_ and Ŝ_V_ accordingly into a histogram (with four bins corresponding to the four buttons). Thus, we obtained a histogram of predicted auditory or visual localization responses for each condition and participant. Based on these histograms we computed the probability of a participant’s counts of localization responses using the multinomial distribution (see Koerding and colleagues [2]). This gives the likelihood of the model given participants’ response data. Assuming independence of experimental conditions, we summed the log likelihoods across conditions.

To obtain maximum likelihood estimates for the parameters of the models (p_common_, σ_P_, σ_A_, σ_V1_ − σ_V2_ for the two levels of visual reliability; formally, the forced-fusion and full-segregation models assume p_common_ = 1 or = 0, respectively), we used a non-linear simplex optimization algorithm as implemented in MATLAB’s fmin search function (MATLAB R2010b). This optimization algorithm was initialized with 200 different parameter settings that were defined based on a prior grid search. We report the results (across-subjects' mean and standard error) from the parameter setting with the highest log likelihood across the 200 initializations (Table 1). This fitting procedure was applied individually to each participant’s data set for the Bayesian Causal Inference, the forced-fusion, and the full-segregation models.

The model fit was assessed by the coefficient of determination R^2^ [40] defined as

$${\text{R}}^{2}\;=\;1-\text{exp}(-\frac{2}{\text{n}}(\text{l}(\hat{ß})-\text{l}(0)))$$

where l($\hat{ß}$) and l(0) denote the log likelihoods of the fitted and the null model, respectively, and n is the number of data points. For the null model, we assumed that an observer randomly chooses one of the four response options, i.e., we assumed a discrete uniform distribution with a probability of 0.25. As in our case the Bayesian Causal Inference model’s responses were discretized to relate them to the four discrete response options, the coefficient of determination was scaled (i.e., divided) by the maximum coefficient (cf. [40]) defined as

$$\text{max}({\text{R}}^{2})\;=\;1-\text{exp}(\frac{2}{\text{n}}\text{l}(0))$$

To identify the optimal model for explaining participants’ data, we compared the candidate models using the Bayesian information criterion (BIC) as an approximation to the model evidence [41]. The BIC depends on both model complexity and model fit. We performed Bayesian model selection [42] at the group level as implemented in SPM8 [43] to obtain the exceedance probability for the candidate models (i.e., the probability that a given model is more likely than any other model given the data).

### MRI Data Acquisition

A 3T Siemens Magnetom Trio MR scanner was used to acquire both T1-weighted anatomical images and T2*-weighted axial echoplanar images with BOLD contrast (gradient echo, parallel imaging using GRAPPA with an acceleration factor of 2, TR = 2,480 ms, TE = 40 ms, flip angle = 90°, FOV = 192 × 192 mm^2^, image matrix 78 × 78, 42 transversal slices acquired interleaved in ascending direction, voxel size = 2.5 × 2.5 × 2.5 mm^3^ + 0.25 mm interslice gap).

In total, 353 volumes times 20 sessions were acquired for the ventriloquist paradigm, 161 volumes times 2–4 sessions for the auditory localizer and 159 volumes times 10–16 sessions for the visual retinotopic localizer resulting in approximately 18 hours of scanning in total per participant assigned over 7–11 days. The first three volumes of each session were discarded to allow for T1 equilibration effects.

### fMRI Data Analysis

**Ventriloquist paradigm.** The fMRI data were analyzed with SPM8 (http://www.fil.ion.ucl.ac.uk/spm) [43]. Scans from each participant were corrected for slice timing, were realigned and unwarped to correct for head motion and spatially smoothed with a Gaussian kernel of 3 mm FWHM. The time series in each voxel was high-pass filtered to 1/128 Hz. All data were analyzed in native participant space. The fMRI experiment was modelled in an event-related fashion with regressors entering into the design matrix after convolving each event-related unit impulse with a canonical hemodynamic response function and its first temporal derivative. In addition to modelling the 32 conditions in our 4 (auditory locations) × 4 (visual locations) × 2 (visual reliability) factorial design, the general linear model included the realignment parameters as nuisance covariates to account for residual motion artefacts. The factor task-relevance (visual versus auditory report) was modelled across sessions.

The parameter estimates pertaining to the canonical hemodynamic response function defined the magnitude of the BOLD response to the audiovisual stimuli in each voxel. For the multivariate decoding analysis, we extracted the parameter estimates of the canonical hemodynamic response function for each condition and session from voxels of the regions of interest (= fMRI voxel response patterns) defined in separate auditory and retinotopic localizer experiments (see below). Each fMRI voxel response pattern for the 64 conditions in our 4 × 4 × 2 × 2 factorial design was based on 11 trials within a particular session. To avoid the effects of image-wide activity changes, each fMRI voxel response pattern was normalized to have mean zero and standard deviation one.

**Decoding of spatial estimates.** To investigate whether and how regions along the auditory and visual spatial processing hierarchy (defined below; cf. Fig 3C) represent spatial estimates of the Causal Inference model, we used a multivariate decoding approach where we decoded each of the four spatial estimates from the regions of interest: (i) the full-segregation visual estimate: Ŝ_V,C = 2_, (ii) the full-segregation auditory estimate: Ŝ_A,C = 2_, (iii) the forced-fusion audiovisual estimate: Ŝ_AV,C = 1_, and (iv) the Bayesian Causal Inference (i.e., model averaging) estimate: Ŝ_A_ & Ŝ_V_, pooled over auditory and visual report (i.e., for each condition we selected the model averaging estimate that needs to be reported in a particular task context). Thus, our decoding approach implicitly assumed that the forced-fusion as well as the auditory and visual estimates under full segregation are computed automatically irrespective of task-context. By contrast, the final auditory or visual Bayesian Causal Inference estimates are flexibly computed depending on the particular task-context according to a decision function such as model averaging. After fitting the Causal Inference model individually to behavioral localization responses (see above), the fitted model predicted these four spatial estimates’ values in 10,000 simulated trials for each of the 64 conditions. The spatial estimates’ values as an index of participants’ perceived location are of a continuous nature. Finally, we summarized the posterior distribution of spatial estimates (i.e., participant’s perceived location) by averaging the values across those 10,000 simulated trials for each of the four spatial estimates separately for each condition and participant. Please note that using the maximum a posteriori estimate as a summary index for the posterior distribution provided nearly equivalent results.

For decoding, we trained a linear support vector regression model (SVR, as implemented in LIBSVM 3.14 [44]) to accommodate the continuous nature of these mean spatial estimates that reflect the perceived signal location for a particular condition and subject. More specifically, we employed a leave “one session” out cross-validation scheme: First, we extracted the voxel response patterns in a particular region of interest (e.g., V1) from the parameter estimate images pertaining to the magnitude of the BOLD response for each condition and session (i.e., 32 conditions × 10 sessions for auditory report + 32 conditions × 10 sessions for visual report = 640 voxel response patterns). For each of the four spatial estimates (e.g., Ŝ_V,C = 2_), we trained one SVR model to learn the mapping from the condition-specific fMRI voxel response patterns (i.e., examples) to the condition-specific spatial estimate’s values (i.e., labels) from all but one session (i.e., 640 − 32 = 608 voxel responses patterns). The model then used this learnt mapping to decode the spatial estimates from the 32 voxel response patterns from the single remaining session. In a leave-one-session-out cross-validation scheme, the training-test procedure was repeated for all sessions. The SVRs’ parameters (C and ν) were optimized using a grid search within each cross-validation fold (i.e., nested cross-validation).

We quantified the decoding accuracies for each of these four spatial estimates in terms of the correlation coefficient between (i) the spatial estimates obtained from the Causal Inference model fitted individually to a participant’s localization responses (i.e., these spatial estimates were used as training labels for fMRI decoding, e.g., Ŝ_V,C = 2_) and (ii) the spatial estimates decoded from fMRI voxel response patterns using SVR. To determine whether the spatial estimates (i.e., labels) can be decoded from the voxel response patterns, we entered the Fisher z-transformed correlation coefficients for each participant into a between-subject one-sample *t*-test and tested whether the across-participants mean correlation coefficient was significantly different from zero separately for the (i) segregated auditory or (ii) visual, (iii) forced-fusion audiovisual, or (iv) auditory and visual Bayesian Causal Inference estimate. As these four spatial estimates were inherently correlated, most regions showed significant positive correlation coefficients for several or even all spatial estimates (see S6 Table). Thus, to determine which of the four spatial estimates was predominantly represented in a region, we computed the exceedance probabilities (i.e., the probability that the correlation coefficient of one spatial estimate is greater than the correlation coefficient of any other spatial estimate) using non-parametric bootstrapping across participants (*N* = 1,000 times). For each bootstrap, we resampled the 5 (= number of participants) individual Fisher z-transformed correlation coefficients with replacement from the set of participants for each of the four spatial estimates and formed the across participants’ mean correlation coefficient for each of the four spatial estimates [45]. In each bootstrap, we then determined which of the four spatial estimates obtained the largest mean correlation coefficient. We repeated this procedure for 1,000 bootstraps. The fraction of bootstraps in which a decoded spatial estimate (e.g., the segregated auditory estimate) had the largest mean correlation coefficient (indexing decoding accuracy) was defined as a spatial estimate’s exceedance probability (Fig 3D). Please note that under the null hypothesis, we would expect that none of the four spatial estimates is related to the voxel response pattern resulting in a uniform distribution of exceedance probabilities for all four spatial estimates (i.e., exceedance probability of 0.25).

**Auditory and visual retinotopic localizer.** Auditory and visual retinotopic localizers were used to define regions of interest along the auditory and visual processing hierarchies in a participant-specific fashion. In the auditory localizer, participants were presented with brief bursts of white noise at −10° or 10° visual angle (duration 500 ms, stimulus onset asynchrony 1 s). In a one-back task, participants indicated via a key press when the spatial location of the current trial was different from the previous trial. 20 s blocks of auditory conditions (i.e., 20 trials) alternated with 13 s fixation periods. The auditory locations were presented in a pseudorandomized fashion to optimize design efficiency. Similar to the main experiment, the auditory localizer sessions were modelled in an event-related fashion with the onset vectors of left and right auditory stimuli being entered into the design matrix after convolution with the hemodynamic response function and its first temporal derivative. Auditory responsive regions were defined as voxels in superior temporal and Heschl’s gyrus showing significant activations for auditory stimulation relative to fixation (*p* < 0.05, family-wise error corrected). Within these regions, we defined primary auditory cortex (A1) based on cytoarchitectonic probability maps [46] and referred to the remainder (i.e., planum temporale and posterior superior temporal gyrus) as higher-order auditory area (hA, see Fig 3C).

Standard phase-encoded retinotopic mapping [47] was used to define visual regions of interest (http://sampendu.wordpress.com/retinotopy-tutorial/). Participants viewed a checkerboard background flickering at 7.5 Hz through a rotating wedge aperture of 70° width (polar angle mapping) or an expanding/contracting ring (eccentricity mapping). The periodicity of the apertures was 42 s. Visual responses were modelled by entering a sine and cosine convolved with the hemodynamic response function as regressors in a general linear model. The preferred polar angle was determined as the phase lag for each voxel, which is the angle between the parameter estimates for the sine and the cosine. The preferred phase lags for each voxel were projected on the reconstructed, inflated cortical surface using Freesurfer 5.1.0 [48]. Visual regions V1–V3, V3AB, and IPS0-IPS4 were defined as phase reversal in angular retinotopic maps. IPS0–4 were defined as contiguous, approximately rectangular regions based on phase reversals along the anatomical IPS [49]. For the decoding analyses, the auditory and visual regions were combined from the left and right hemispheres.

## Supporting Information

S1 Data Zip file containing datasets underlying Figs. 2 and 3D. The data is stored in MATLAB structures.
(ZIP)

S1 Fig BOLD activation (across-subjects mean ± SEM parameter estimates) as a function of visual reliability (VR) and visual location (V) (left subpanel), visual reliability and auditory location (A) (middle subpanel), and visual reliability and task-relevance (T) (right subpanel), separately for the regions of interest in the left (solid) and right (dotted) hemisphere. Using singular value decomposition, parameter estimates were pooled across the voxels of each region of interest by averaging the first eigenvariate of voxel response patterns across the ten replications of each parameter estimate. Significant effects (*p* < 0.05, cf. S2 Table) are noted at the top of each subpanel for the left (L) and right (R) hemisphere.
(TIF)

S1 Table Statistical results of main and interaction effects of the 4 (visual location, V) × 4 (auditory location, A) × 2 (task-relevance, T) × 2 (visual reliability, VR) design on the accuracy of behavioral localization responses. *p* < 0.05 in bold.
(DOCX)

S2 Table Statistical results of main and interaction effects of the factors task-relevance (T), visual reliability (VR), visual signal location (V), and auditory signal location (A) on the mean activation in the left (L) and right (R) regions of interest. Significant effects (*p* < 0.05) are marked in red. Degrees of freedom (df1, df2) for each effect are indicated below F and *p* values.
(DOCX)

S3 Table Individual R^2^ and relative Bayesian information criterion of the Causal Inference model using the ‚model averaging’ (MA), ‚model selection’ (MS), and 'probability matching' (PM) decision strategies. R^2^ = coefficient of determination. relBIC = Bayesian information criterion (BIC = LL − 0.5 M ln(N), LL = log likelihood, M = number of parameters, N = number of data points) of a model relative to a participant’s best model (smaller relBIC indicates that a model provides a better explanation of a participant’s data).
(DOCX)

S4 Table Model parameters, R^2^ (across-subjects mean ± SEM) and group Bayesian information iriterion of the Bayesian Causal Inference model for the three different decision strategies. Note: p_C_ = prior common-source probability. σ_P_ = standard deviation of the spatial prior (in °). σ_A_ = standard deviation of the auditory likelihood (in °). σ_V_ = standard deviation of the visual likelihood at two levels of visual reliability (1 = high, 2 = low) (in °). R^2^ = coefficient of determination. relBIC_Group_ = Bayesian information criterion at the group level, i.e., subject-specific BICs summed over all subjects (BIC = LL − 0.5 M ln(N), LL = log likelihood, M = number of parameters, N = number of data points) of a model relative to the best “model averaging” model (n.b. a smaller relBIC_Group_ indicates that a model provides a better explanation of our data). EP = exceedance probability, i.e., probability that a model is more likely than any other model from the random effects model comparison (see Materials and Methods section in main paper).
(DOCX)

S5 Table Posterior common-source probability of the fitted Causal Inference model (across-subjects mean ± SEM; “model averaging”) as a function of absolute audiovisual disparity and visual reliability. Note: Visual reliability is the inverse of the visual variance determined by the standard deviation of the visual cloud of dots (i.e., 2° = high, 14° = low visual reliability). Both visual reliability and audiovisual disparity are specified in degree visual angle as units.
(DOCX)

S6 Table Decoding accuracies (across-subjects' mean correlation) for the four spatial estimates of the Bayesian Causal Inference model (i.e., model averaging) in the regions of interest. Significant decoding accuracies (across-subjects' one sample *t*-tests against zero of Fisher z-transformed correlation coefficients) are marked by asterisks (*p < 0.05; **p < 0.01; ***p < 0.001).
(DOCX)

S1 Text Supporting methods, results, and discussion. (DOCX)

## Abbreviations

BOLD: blood-oxygen-level dependent
fMRI: functional magnetic resonance imaging
MR: magnetic resonance
SEM: standard error of the mean
SVR: support vector regression
