# Supplementary figures and images for: Cortical Hierarchies Perform Bayesian Causal Inference in Multisensory Perception
Source: PLoS Biol. 2015 Feb 24;13(2):e1002073. doi: 10.1371/journal.pbio.1002073 (PMC4339735; doi:10.1371/journal.pbio.1002073)

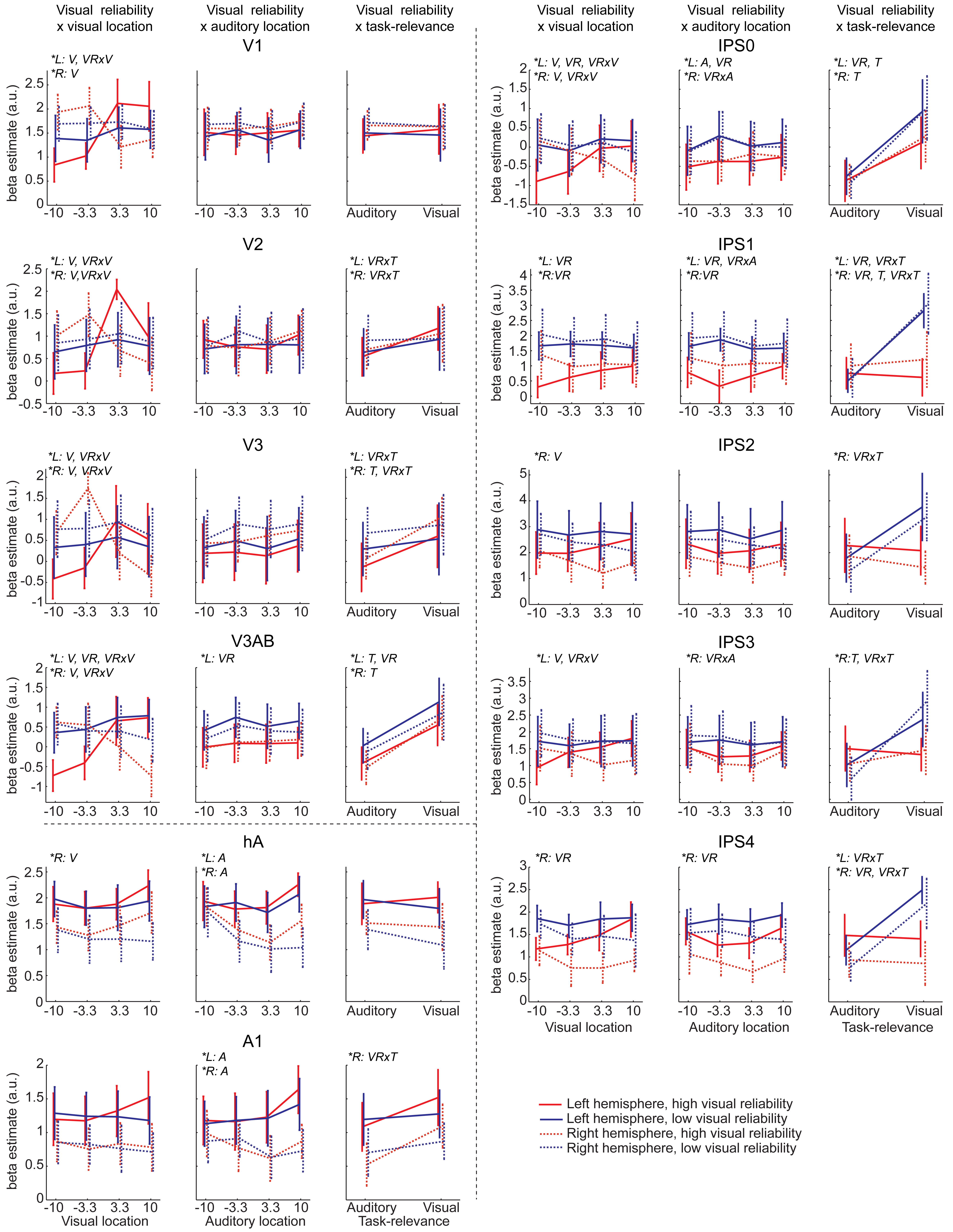

Supplement: S1 Fig — Using singular value decomposition, parameter estimates were pooled across the voxels of each region of interest by averaging the first eigenvariate of voxel response patterns across the ten replications of each parameter estimate. Significant effects (p < 0.05, cf. S2 Table) are noted at the top of each subpanel for the left (L) and right (R) hemisphere. (TIF) [file pbio.1002073.s002.tif]
