# Supplementary material for: Cortical Hierarchies Perform Bayesian Causal Inference in Multisensory Perception
Source: PLoS Biol. 2015 Feb 24;13(2):e1002073. doi: 10.1371/journal.pbio.1002073 (PMC4339735; doi:10.1371/journal.pbio.1002073)
Supplement: S1 Table — p < 0.05 in bold. (DOCX) [file pbio.1002073.s003.docx]

| **Table S1**. Statistical results of main and interaction effects of the 4 (visual location, V) x 4 (auditory location, A) x 2 (task-relevance, T) x 2 (visual reliability, VR) design on the accuracy of behavioral localization responses. | | | | |
| --- | --- | --- | --- | --- |
|  | F | df1 | df2 | p |
| T | 11.28 | 1 | 4 | **0.028** |
| VR | 11.62 | 1 | 4 | **0.027** |
| V | 0.65 | 3 | 12 | 0.598 |
| A | 10.80 | 3 | 12 | **0.001** |
| TxVR | 25.86 | 1 | 4 | **0.007** |
| TxV | 0.45 | 3 | 12 | 0.724 |
| TxA | 10.67 | 3 | 12 | **0.001** |
| VRxV | 0.61 | 3 | 12 | 0.619 |
| VRxA | 1.93 | 3 | 12 | 0.179 |
| VxA | 11.31 | 9 | 36 | **<0.001** |
| TxVRxV | 1.31 | 3 | 12 | 0.317 |
| TxVRxA | 1.14 | 3 | 12 | 0.373 |
| TxVxA | 13.41 | 9 | 36 | **<0.001** |
| VRxVxA | 14.82 | 9 | 36 | **<0.001** |
| TxVRxVxA | 11.32 | 9 | 36 | **<0.001** |
