# Supplementary material for: Cortical Hierarchies Perform Bayesian Causal Inference in Multisensory Perception
Source: PLoS Biol. 2015 Feb 24;13(2):e1002073. doi: 10.1371/journal.pbio.1002073 (PMC4339735; doi:10.1371/journal.pbio.1002073)
Supplement: S2 Table — Significant effects (p < 0.05) are marked in red. Degrees of freedom (df1, df2) for each effect are indicated below F and p values. (DOCX) [file pbio.1002073.s004.docx]

| **Table S2.** Statistical results of main and interaction effects of the factors task-relevance (T), visual reliability (VR), visual signal location (V) and auditory signal location (A) on the mean activation in the left (L) and right (R) regions of interest. | | | | | | | | | | | | | | | | | | | | | | | | | | | | | | | | | | | | | | | | | |
| --- | --- | --- | --- | --- | --- | --- | --- | --- | --- | --- | --- | --- | --- | --- | --- | --- | --- | --- | --- | --- | --- | --- | --- | --- | --- | --- | --- | --- | --- | --- | --- | --- | --- | --- | --- | --- | --- | --- | --- | --- | --- |
|  |  | T | | VR | | V | | A | | TxVR | | TxV | | TxA | | | VRxV | | | VRxA | | | VxA | | | T  xVR  xV | | | | T  xVR  xA | | | T  xV  xA | | VR  xV  xA | | | | T  xVR  xV  xA | | |
|  |  | F | p | F | p | F | p | F | p | F | p | F | p | | F | p | | F | p | | F | p | | F | p | | F | p | | F | p | | F | p | | F | p | | | F | p |
|  |  | 1,4 | | 1,4 | | 3,12 | | 3,12 | | 1,4 | | 13,12 | | 3,12 | | | 3,12 | | | 3,12 | | | 9,36 | | | 3,12 | | | 3,12 | | | 9,36 | | | 9,36 | | | 9,36 | | | |
| V1 | L | <0.1 | 0.900 | 0.1 | 0.801 | 18.0 | <0.001 | 0.7 | 0.543 | 2.3 | 0.205 | 0.6 | 0.629 | | 0.9 | 0.480 | | 5.6 | 0.012 | | 0.7 | 0.583 | | 0.7 | 0.721 | | 0.7 | 0.559 | | 2.4 | 0.114 | | 0.7 | 0.667 | | 0.9 | 0.496 | | | 0.3 | 0.959 |
|  | R | <0.1 | 0.878 | 0.3 | 0.617 | 4.4 | 0.027 | 1.6 | 0.247 | 0.3 | 0.601 | 0.1 | 0.939 | | 0.8 | 0.506 | | 3.4 | 0.052 | | 0.5 | 0.699 | | 0.6 | 0.768 | | 0.3 | 0.852 | | 1.3 | 0.317 | | 0.6 | 0.756 | | 0.9 | 0.531 | | | 1.0 | 0.468 |
| V2 | L | 5.2 | 0.084 | 0.1 | 0.768 | 13.7 | <0.001 | 0.8 | 0.521 | 14.2 | 0.020 | 2.7 | 0.095 | | 2.2 | 0.142 | | 9.2 | 0.002 | | 2.6 | 0.104 | | 1.3 | 0.276 | | 1.6 | 0.238 | | 1.6 | 0.233 | | 0.9 | 0.569 | | 1.3 | 0.288 | | | 1.0 | 0.459 |
|  | R | 1.0 | 0.381 | 0.1 | 0.744 | 8.8 | 0.002 | 1.9 | 0.190 | 15.5 | 0.017 | 0.5 | 0.674 | | 1.5 | 0.264 | | 4.5 | 0.025 | | 2.2 | 0.146 | | 1.1 | 0.359 | | 2.9 | 0.078 | | 3.0 | 0.071 | | 0.6 | 0.820 | | 1.2 | 0.329 | | | 1.3 | 0.271 |
| V3 | L | 3.0 | 0.160 | 3.6 | 0.131 | 7.0 | 0.006 | 0.8 | 0.528 | 40.2 | 0.003 | 1.9 | 0.184 | | 0.8 | 0.507 | | 4.7 | 0.022 | | 0.5 | 0.721 | | 0.7 | 0.714 | | 0.2 | 0.924 | | 0.7 | 0.572 | | 0.9 | 0.498 | | 0.6 | 0.794 | | | 1.2 | 0.355 |
|  | R | 14.5 | 0.019 | 0.7 | 0.465 | 14.8 | <0.001 | 2.8 | 0.087 | 24.2 | 0.008 | 1.7 | 0.210 | | 1.0 | 0.415 | | 10.2 | 0.001 | | 1.0 | 0.409 | | 0.9 | 0.534 | | 2.9 | 0.082 | | 0.7 | 0.578 | | 0.7 | 0.677 | | 1.2 | 0.337 | | | 0.8 | 0.612 |
| V3AB | L | 18.4 | 0.013 | 11.7 | 0.027 | 16.1 | <0.001 | 1.1 | 0.392 | 0.6 | 0.493 | 1.0 | 0.411 | | 1.5 | 0.254 | | 6.6 | 0.007 | | 2.3 | 0.125 | | 1.2 | 0.334 | | 0.2 | 0.895 | | 1.1 | 0.400 | | 1.4 | 0.242 | | 0.7 | 0.743 | | | 1.7 | 0.134 |
|  | R | 21.7 | 0.010 | 2.5 | 0.190 | 8.9 | 0.002 | 2.3 | 0.128 | 2.8 | 0.170 | 0.9 | 0.467 | | 1.6 | 0.249 | | 5.7 | 0.011 | | 2.0 | 0.166 | | 0.9 | 0.533 | | 0.9 | 0.467 | | 0.5 | 0.685 | | 1.7 | 0.121 | | 0.9 | 0.532 | | | 2.4 | 0.032 |
| IPS-0 | L | 14.4 | 0.019 | 9.1 | 0.039 | 11.4 | 0.001 | 3.6 | 0.046 | 6.6 | 0.062 | 1.0 | 0.409 | | 1.5 | 0.257 | | 5.1 | 0.017 | | 1.8 | 0.207 | | 2.1 | 0.062 | | 0.9 | 0.483 | | 0.6 | 0.648 | | 2.0 | 0.069 | | 0.9 | 0.524 | | | 1.3 | 0.265 |
|  | R | 16.7 | 0.015 | 3.6 | 0.130 | 9.4 | 0.002 | 1.2 | 0.361 | 2.4 | 0.194 | 0.6 | 0.634 | | 3.5 | 0.051 | | 3.8 | 0.039 | | 4.5 | 0.025 | | 0.9 | 0.576 | | 1.6 | 0.231 | | 1.1 | 0.386 | | 0.7 | 0.698 | | 0.8 | 0.577 | | | 1.8 | 0.111 |
| IPS-1 | L | 5.5 | 0.079 | 10.5 | 0.032 | 1.5 | 0.269 | 1.1 | 0.369 | 20.9 | 0.010 | 0.8 | 0.501 | | 0.6 | 0.598 | | 2.2 | 0.146 | | 5.3 | 0.015 | | 0.6 | 0.788 | | 1.2 | 0.336 | | 2.3 | 0.129 | | 1.5 | 0.178 | | 1.3 | 0.271 | | | 0.9 | 0.515 |
|  | R | 7.8 | <0.050 | 7.8 | 0.049 | 3.3 | 0.059 | 0.9 | 0.484 | 19.9 | 0.011 | 0.5 | 0.721 | | 0.8 | 0.514 | | 0.2 | 0.922 | | 1.2 | 0.342 | | 1.0 | 0.439 | | 1.0 | 0.408 | | 1.3 | 0.308 | | 2.1 | 0.051 | | 1.0 | 0.466 | | | 0.6 | 0.817 |
| IPS-2 | L | 1.6 | 0.277 | 2.8 | 0.170 | 0.6 | 0.646 | 1.2 | 0.364 | 5.6 | 0.078 | 0.4 | 0.734 | | 0.1 | 0.935 | | 1.1 | 0.402 | | 1.2 | 0.365 | | 0.9 | 0.522 | | 1.5 | 0.262 | | 1.2 | 0.340 | | 1.7 | 0.134 | | 1.4 | 0.244 | | | 1.2 | 0.331 |
|  | R | 1.8 | 0.255 | 4.6 | 0.099 | 5.1 | 0.016 | 1.3 | 0.327 | 14.4 | 0.019 | 0.7 | 0.574 | | 0.8 | 0.540 | | 0.5 | 0.666 | | 0.9 | 0.466 | | 1.0 | 0.458 | | 0.6 | 0.600 | | 1.6 | 0.233 | | 1.8 | 0.096 | | 1.3 | 0.279 | | | 1.9 | 0.079 |
| IPS-3 | L | 2.2 | 0.208 | 0.7 | 0.443 | 3.8 | 0.039 | 1.3 | 0.312 | 4.8 | 0.093 | 1.1 | 0.400 | | 0.9 | 0.481 | | 4.9 | 0.019 | | 1.8 | 0.201 | | 0.9 | 0.546 | | 0.9 | 0.457 | | 2.7 | 0.092 | | 1.2 | 0.331 | | 1.4 | 0.241 | | | 0.6 | 0.764 |
|  | R | 7.8 | <0.050 | 3.1 | 0.152 | 2.8 | 0.085 | 1.5 | 0.271 | 19.5 | 0.012 | 1.0 | 0.410 | | 2.4 | 0.121 | | 0.2 | 0.904 | | 4.7 | 0.021 | | 1.3 | 0.274 | | 0.3 | 0.837 | | 0.0 | 0.991 | | 0.9 | 0.516 | | 1.8 | 0.097 | | | 1.4 | 0.223 |
| IPS-4 | L | 2.2 | 0.212 | 6.1 | 0.069 | 3.4 | 0.053 | 1.3 | 0.332 | 9.2 | 0.039 | 0.3 | 0.798 | | 0.5 | 0.676 | | 2.0 | 0.169 | | 2.7 | 0.093 | | 1.6 | 0.143 | | 0.8 | 0.533 | | 2.2 | 0.135 | | 1.4 | 0.232 | | 1.2 | 0.302 | | | 0.7 | 0.735 |
|  | R | 2.1 | 0.222 | 12.4 | 0.025 | 3.2 | 0.063 | 0.9 | 0.488 | 13.9 | 0.020 | 0.5 | 0.695 | | 2.2 | 0.140 | | 0.2 | 0.898 | | 1.9 | 0.180 | | 1.5 | 0.182 | | 0.7 | 0.594 | | 0.3 | 0.836 | | 0.7 | 0.746 | | 1.7 | 0.128 | | | 0.9 | 0.529 |
| hA | L | <0.1 | 0.918 | 0.8 | 0.419 | 3.3 | 0.057 | 5.0 | 0.018 | 0.9 | 0.406 | 0.7 | 0.572 | | 1.2 | 0.352 | | 1.5 | 0.262 | | 1.2 | 0.357 | | 0.6 | 0.816 | | 0.7 | 0.573 | | 0.0 | 0.993 | | 1.6 | 0.144 | | 1.3 | 0.266 | | | 1.2 | 0.307 |
|  | R | 1.1 | 0.356 | 4.0 | 0.116 | 3.9 | 0.038 | 12.8 | <0.001 | 1.0 | 0.374 | 0.8 | 0.501 | | 2.6 | 0.104 | | 1.4 | 0.288 | | 2.0 | 0.172 | | 1.5 | 0.190 | | 0.3 | 0.830 | | 0.6 | 0.613 | | 1.4 | 0.237 | | 1.6 | 0.162 | | | 1.7 | 0.138 |
| A1 | L | 3.3 | 0.144 | 1.2 | 0.326 | 1.3 | 0.309 | 8.9 | 0.002 | 3.8 | 0.121 | 1.1 | 0.405 | | 1.4 | 0.299 | | 2.5 | 0.113 | | 0.7 | 0.596 | | 0.3 | 0.984 | | 1.4 | 0.278 | | 0.2 | 0.901 | | 1.0 | 0.447 | | 0.9 | 0.500 | | | 1.5 | 0.188 |
|  | R | 5.8 | 0.073 | 0.3 | 0.636 | 0.9 | 0.481 | 4.1 | 0.033 | 10.5 | 0.032 | 0.1 | 0.962 | | 2.2 | 0.145 | | 0.3 | 0.853 | | 1.5 | 0.259 | | 0.6 | 0.793 | | 0.1 | 0.986 | | 1.6 | 0.238 | | 0.7 | 0.716 | | 0.8 | 0.593 | | | 1.1 | 0.397 |
