# Supplementary material for: Cortical Hierarchies Perform Bayesian Causal Inference in Multisensory Perception
Source: PLoS Biol. 2015 Feb 24;13(2):e1002073. doi: 10.1371/journal.pbio.1002073 (PMC4339735; doi:10.1371/journal.pbio.1002073)
Supplement: S3 Table — R2 = coefficient of determination. relBIC = Bayesian information criterion (BIC = LL − 0.5 M ln(N), LL = log likelihood, M = number of parameters, N = number of data points) of a model relative to a participant’s best model (smaller relBIC indicates that a model provides a better explanation of a participant’s data). (DOCX) [file pbio.1002073.s005.docx]

| **Table S3.** Individual R^2^ and relative Bayesian Information Criterion of the Causal Inference model using the ‚model averaging‘ (MA), ‚model selection‘ (MS) and ‚probability matching‘ (PM) decision strategies. | | | | | | |
| --- | --- | --- | --- | --- | --- | --- |
|  | MA | | MS | | PM | |
| Subject | R^2^ | relBIC | R^2^ | relBic | R^2^ | relBIC |
| 1 | 92.9 | 102.3 | 93.7 | 0 | 93.3 | 50.6 |
| 2 | 77.3 | 35.4 | 77.4 | 31 | 77.9 | 0 |
| 3 | 89.1 | 0 | 84.6 | 390 | 86.6 | 223.2 |
| 4 | 80.9 | 0 | 79.6 | 69.2 | 80.7 | 12.0 |
| 5 | 71.9 | 0 | 70.1 | 92.2 | 70.2 | 88.7 |
