# Supplementary material for: Cortical Hierarchies Perform Bayesian Causal Inference in Multisensory Perception
Source: PLoS Biol. 2015 Feb 24;13(2):e1002073. doi: 10.1371/journal.pbio.1002073 (PMC4339735; doi:10.1371/journal.pbio.1002073)
Supplement: S4 Table — Note: pC = prior common-source probability. σP = standard deviation of the spatial prior (in °). σA = standard deviation of the auditory likelihood (in °). σV = standard deviation of the visual likelihood at two levels of visual reliability (1 = high, 2 = low) (in °). R2 = coefficient of determination. relBICGroup = Bayesian information criterion at the group level, i.e., subject-specific BICs summed over all subjects (BIC = LL − 0.5 M ln(N), LL = log likelihood, M = number of parameters, N = number of data points) of a model relative to the best “model averaging” model (n.b. a smaller relBICGroup indicates that a model provides a better explanation of our data). EP = exceedance probability, i.e., probability that a model is more likely than any other model from the random effects model comparison (see Materials and Methods section in main paper). (DOCX) [file pbio.1002073.s006.docx]

| **Table S4.** Model parameters, R^2^ (across-subjects mean ± SEM) and Group Bayesian Information Criterion of the Bayesian Causal Inference model for the three different decision strategies. | | | | | | | | |
| --- | --- | --- | --- | --- | --- | --- | --- | --- |
| Model | p_C_ | σ_P_ | σ_A_ | σ_V1_ | σ_V2_ | R^2^ | relBIC_Group_ | EP |
| Model averaging | 0.48+0.10 | 14.9+3.8 | 17.1+7.0 | 3.8+0.5 | 8.3+0.8 | 82.4+3.8 | 0 | 0.700 |
| Model selection | 0.43+0.08 | 11.2+2.0 | 19.1+8.2 | 3.9+0.5 | 9.6+1.3 | 81.1+3.9 | 444.7 | 0.155 |
| Probability matching | 0.29+0.07 | 11.6+2.1 | 19.3+8.3 | 3.9+0.5 | 8.4+1.0 | 81.7+3.9 | 236.7 | 0.145 |
