# Supplementary material for: Cortical Hierarchies Perform Bayesian Causal Inference in Multisensory Perception
Source: PLoS Biol. 2015 Feb 24;13(2):e1002073. doi: 10.1371/journal.pbio.1002073 (PMC4339735; doi:10.1371/journal.pbio.1002073)
Supplement: S5 Table — Note: Visual reliability is the inverse of the visual variance determined by the standard deviation of the visual cloud of dots (i.e., 2° = high, 14° = low visual reliability). Both visual reliability and audiovisual disparity are specified in degree visual angle as units. (DOCX) [file pbio.1002073.s007.docx]

| **Table S5.** Posterior common-source probability of the fitted Causal Inference model (across-subjects mean ± SEM; ‘model averaging’) as a function of absolute audiovisual disparity and visual reliability. | | | | | |
| --- | --- | --- | --- | --- | --- |
|  |  | Absolute audiovisual disparity (°) | | | |
|  |  | 0 | 6.6 | 13.3 | 20 |
| Visual reliability (°) | 2 | 0.50±0.19 | 0.44±0.19 | 0.33±0.17 | 0.23±0.16 |
|  | 14 | 0.47±0.19 | 0.43±0.19 | 0.35±0.18 | 0.27±0.17 |
