# Supplementary material for: Cortical Hierarchies Perform Bayesian Causal Inference in Multisensory Perception
Source: PLoS Biol. 2015 Feb 24;13(2):e1002073. doi: 10.1371/journal.pbio.1002073 (PMC4339735; doi:10.1371/journal.pbio.1002073)
Supplement: S6 Table — Significant decoding accuracies (across-subjects' one sample t-tests against zero of Fisher z-transformed correlation coefficients) are marked by asterisks (*p < 0.05; **p < 0.01; ***p < 0.001). (DOCX) [file pbio.1002073.s008.docx]

| **Table S6.** Decoding accuracies (across-subjects mean correlation) for the four spatial estimates of the Bayesian Causal Inference model (i.e., model averaging) in the regions of interest. | | | | | | | | | | | | |
| --- | --- | --- | --- | --- | --- | --- | --- | --- | --- | --- | --- | --- |
|  | **V1** | **V2** | **V3** | **V3AB** | **IPS-0** | **IPS-1** | **IPS-2** | **IPS-3** | **IPS-4** | **hA** | **A1** | |
| $\hat{S}\text{A}$ & $\hat{S}\text{V}$ | 0.60** | 0.57** | 0.62** | 0.58** | 0.54** | 0.36** | 0.41*** | 0.44*** | 0.37*** | 0.32** | 0.15** |  |
| $\hat{S}\text{AV,C=1}$ | 0.74*** | 0.70*** | 0.74*** | 0.69*** | 0.61** | 0.39** | 0.45*** | 0.43** | 0.32** | 0.27* | 0.11* |  |
| $\hat{S}\text{V,C=2}$ | 0.79*** | 0.75*** | 0.79*** | 0.71*** | 0.63** | 0.38** | 0.44*** | 0.39** | 0.29** | 0.17 | 0.06 |  |
| $\hat{S}\text{A,C=2}$ | 0.02 | -0.02 | 0.05 | 0.05 | 0.07* | 0.08* | 0.09* | 0.10 | 0.12* | 0.40** | 0.18** |  |
