# Supplementary material for: Cortical Hierarchies Perform Bayesian Causal Inference in Multisensory Perception
Source: PLoS Biol. 2015 Feb 24;13(2):e1002073. doi: 10.1371/journal.pbio.1002073 (PMC4339735; doi:10.1371/journal.pbio.1002073)
Supplement: S1 Text — (DOCX) [file pbio.1002073.s009.docx]

**Supporting Methods, Results and Discussion**

**Behavioral Analysis and Results of Localization Accuracy**

The localization accuracies for each of the 64 conditions were entered into a 4 (visual location) x 4 (auditory location) x 2 (visual reliability) x 2 (task-relevance) repeated measures ANOVA. The statistical results are reported in detail in supporting Table S1. Consistent with the response deviation profile shown in Figure 2, participants were more accurate when locating the visual than the auditory signals (i.e., main effect of task-relevance). Likewise, localization accuracy was higher for reliable relative to unreliable visual signals. Most importantly, we observed a significant interaction between visual and auditory location on localization accuracy. This accuracy profile reflects the fact that participants were more accurate when locating spatially congruent relative to spatially discrepant audiovisual signals. In fact, the visual spatial bias on the perceived auditory location that is illustrated in Figure 2 is also reflected in reduced accuracy for spatially discrepant audiovisual signals (for further results see supporting Table S1).

**Conventional fMRI Analysis in the Regions of Interest**

We complemented the analysis based on the Bayesian Causal Inference model with a conventional fMRI analysis in the regions of interest along the visual and auditory processing streams. As described in the main paper, the data were preprocessed and analyzed in SPM8. The subject-specific general linear model included the 32 conditions in our 4 (auditory locations) x 4 (visual locations) x 2 (visual reliability) factorial design and the realignment parameters as nuisance covariates in a session-specific fashion. The factor task-relevance (visual vs. auditory report) was modelled across sessions (i.e., 64 conditions in total). From our 20 scanning sessions (i.e., 10 sessions for visual report and 10 sessions for auditory report), we thus obtained 10 parameter estimates pertaining to the canonical hemodynamic response function for each condition (i.e., 10 sessions x 64 conditions = 640 parameter estimate images in total). To provide a summary index for the condition-specific BOLD-response in each region, we performed a singular value decomposition on the voxel response pattern x session matrix and averaged the values of the 1st eigenvariate for each condition separately for each region of interest. These regional response indices for the 64 conditions and for each subject were entered into a 4 (visual location) x 4 (auditory location) x 2 (visual reliability) x 2 (task-relevance) 2nd level between-subject repeated measures ANOVA independently for each region of interest. The F- and p-vaIues for the main effects and interactions are reported in supporting Table S2. The activation profiles across the different regions of interest are shown in supporting Figure S1.

As expected from the retinotopic organization of visual areas, we observed a main effect of visual location in particular in low-level visual areas. Visual signals in the left spatial hemifield increased activations in right V1-IPS0 and signals in the right hemifield in left V1-IPS0. Further, this lateralized response profile was more pronounced for visual signals that were highly reliable (i.e., an interaction of visual location and visual reliability).

Even though this characteristic response profile was also observed in lower intraparietal regions (IPS0), higher-order visual and parietal areas were increasingly influenced by task context, i.e., visual vs. auditory report. In IPS1-4, we observed a significant cross-over interaction between task-relevance and visual reliability. For auditory report, activation increased for high relative to low visual reliability. By contrast, for visual report, activation increased for low relative to high visual reliability. This response profile most likely reflects the demands on attentional and cognitive control resources, which are high (i) when the visual signal is degraded and needs to be reported and (ii) when the visual signal is reliable, but is task-irrelevant and needs to be ignored.

In line with the auditory system’s predominant contralateral processing of spatial signals [[1](#_ENREF_1)], auditory regions (hA, A1) showed activation increases for contra- relative to ipsilateral auditory signals. As previously shown [[2](#_ENREF_2),[3](#_ENREF_3)], we also observed a small influence of visual location on activations in higher auditory regions (hA) encompassing the planum temporale. In addition, we observed an interaction between visual reliability and auditory location in IPS0, IPS1 and IPS3. These audiovisual integration effects in higher-order IPS areas have only been inconsistently reported in the literature [[2](#_ENREF_2),[3](#_ENREF_3),[4](#_ENREF_4)]. For instance, Bonath and colleagues [[2](#_ENREF_2)] reported activations for spatial ventriloquism in planum temporale, superior temporal sulcus, posterior cingulate and medial and superior frontal gyrus. By contrast, Bischoff and colleagues [[4](#_ENREF_4)] reported activations also in parietal and insular areas. Yet, it is important to appreciate that previous studies differed from the current one in several ways. First of all, they employed a different analysis approach and directly compared trials with and without ventriloquist illusion. In other words, for each trial, they determined whether the ventriloquist illusion, i.e., a visual bias on the perceived auditory location, emerged. Second, they used a more constrained experimental design with only few conditions to maximize design efficiency for this statistical comparison (i.e., trials with vs. without ventriloquist illusion). By contrast, our design included many more conditions optimized for identifying the processes underlying Bayesian Causal Inference. For instance, our experimental design included trials with large spatial disparities where audiovisual integration was attenuated. Further, we manipulated the task-relevance of the auditory and visual signals, which is important to determine spatial estimates of ‘Bayesian Causal Inference’. Finally, we also manipulated visual reliability to characterize reliability-weighted integration.

Perhaps surprisingly, we did not observe a direct interaction between auditory and visual location that may have been expected based on the classical spatial principle of multisensory integration [[5](#_ENREF_5)]. In other words, one may have expected a so-called ‘congruency’ effect that would have emerged as an interaction between auditory and visual locations in our design specification. Yet, as our 4 (visual location) x 4 (auditory location) design included only 4 congruent, yet 12 incongruent conditions, it was not optimized to reveal audiovisual congruency effects. Moreover, in inter-sensory selective attention tasks, congruency effects tend to be more pronounced when the interfering, task-irrelevant stimulus is highly reliable (see Noppeney and colleagues [[6](#_ENREF_6)]). Yet, in 75% of the trials in our experimental design the incongruent interfering stimulus was an unreliable auditory (50%) or unreliable visual (25%) signal.

In summary, the results of the conventional fMRI analysis converge with those of our model-based analysis at two levels: First, both conventional and model-based analyses emphasize that low-level sensory areas are predominantly driven by their preferred sensory signals. Thus, regional BOLD-signal indices (i.e., conventional analysis) were influenced by visual location in low-level visual areas and by auditory location in primary auditory areas. Conversely, the Bayesian Causal Inference analysis demonstrated that primary sensory areas predominantly represent the spatial estimate under the assumption of full segregation (e.g., the visual spatial estimate in visual areas). Second, both analyses suggest that higher-order parietal areas are influenced by task context, i.e., whether auditory or visual location should be reported. The conventional analysis demonstrated that the BOLD-response indices in parietal areas depended directly or in interaction with reliability on task-context. Likewise, the model-based analysis revealed that in particular IPS3-4 encoded the Bayesian Causal Inference estimate that combines the forced-fusion and the full-segregation estimates of the *task-relevant* signals. Thus, the Bayesian Causal Inference analysis also showed that the spatial representations in IPS depended on whether the auditory or visual location should be reported. Critically, however, the model-based analysis goes beyond the conventional fMRI analysis by characterizing the spatial representations encoded in voxel response patterns and directly relating those to hidden variables in the Bayesian Causal Inference Model. It provides a more fine-grained analysis of the spatial representations encoded in different areas along the visual and auditory processing streams. Thus, the model-based analysis provides a deeper understanding of how the brain performs Bayesian Causal Inference.

**Model-Based Analysis with Different Decision Functions**

To provide a final estimate of the auditory location, the brain can combine the estimates under the two causal structures using various decision functions. In addition to ‘model averaging’ presented in the main text, we considered two additional decision functions (for details see [[7](#_ENREF_7)]): According to the ‘model selection’ strategy, the brain reports the auditory ($\hat{S}\text{A}$) or visual ($\hat{S}\text{V}$) spatial estimate selectively from the more likely causal structure (for definition of $\hat{S}\text{AV,C=1}$, $\hat{S}\text{A,C=2 }$, $\hat{S}\text{V,C=2}$ and $\text{p(C=1|}x\text{A},x\text{V}\text{)}$) see equations (1)-(3) in the Materials and Methods of the main text).

$\hat{S}\text{A }\text{= }\left\{ \begin{aligned} \hat{S}\text{AV,C=1 }\text{if}\text{ }\text{p(C}\text{ }\text{=}\text{ }\text{1|}x\text{A},x\text{V}\text{)}> 0.5 \\ \hat{S}\text{A,C=2 }\text{ }\text{if}\text{ }\text{p(C}\text{ }\text{=}\text{ }\text{1|}x\text{A},x\text{V}\text{)}\leq0.5 \end{aligned} \right.$ (6)

$\hat{S}\text{V }\text{= }\left\{ \begin{aligned} \hat{S}\text{AV,C=1 }\text{if}\text{ }\text{p(C}\text{ }\text{=}\text{ }\text{1|}x\text{A},x\text{V}\text{)}> 0.5 \\ \hat{S}\text{V,C=2 }\text{ }\text{if}\text{ }\text{p(C}\text{ }\text{=}\text{ }\text{1|}x\text{A},x\text{V}\text{)}\leq0.5 \end{aligned} \right.$ (7)

According to ‘probability matching’, the brain reports the spatial estimate of one causal structure stochastically selected in proportion to its posterior probability.

$\hat{S}\text{A }\text{= }\left\{ \begin{aligned} \hat{S}\text{AV,C=1}\text{ if}\text{ }\text{p(C}\text{ }\text{=}\text{ }\text{1|}x\text{A},x\text{V}\text{)}> \alpha, \alpha\sim U\left( 0,1 \right) \\ \hat{S}\text{A,C=2}\text{ }\text{ if}\text{ }\text{p(C}\text{ }\text{=}\text{ }\text{1|}x\text{A},x\text{V}\text{)}\leq\alpha, \alpha\sim U\left( 0,1 \right) \end{aligned} \right.$ (8)

$\hat{S}\text{V }\text{= }\left\{ \begin{aligned} \hat{S}\text{AV,C=1}\text{ if}\text{ }\text{p(C}\text{ }\text{=}\text{ }\text{1|}x\text{A},x\text{V}\text{)}> \alpha, \alpha\sim U\left( 0,1 \right) \\ \hat{S}\text{V,C=2}\text{ }\text{ }\text{if}\text{ }\text{p(C}\text{ }\text{=}\text{ }\text{1|}x\text{A},x\text{V}\text{)}\leq\alpha, \alpha\sim U\left( 0,1 \right) \end{aligned} \right.$ (9)

Even though probability matching is sub-optimal, humans have been shown to use this strategy in a variety of cognitive tasks (e.g., [[8](#_ENREF_8)]). In particular, a recent study suggested that human observers use probability matching during audiovisual spatial localization [[7](#_ENREF_7)]. The ‘model averaging’, ‘model selection’, ‘probability matching’ models were fit to the participants’ localization responses. Supporting Table S3 shows the R^2^ and BIC values for each of the five participants. Model averaging was the most likely decision strategy in three participants, model selection in one participant and probability matching also in one participant. This inter-subject variability converges with previous reports that human observers consistently adhere to different decision strategies [[7](#_ENREF_7)]. At the group level (see supporting Table S4), model averaging outperformed probability matching and model selection both in terms of the overall BIC (i.e., fixed effects analysis) and the exceedance probability (i.e., random effects analysis). In the main paper, we therefore focused on the model averaging strategy. However, the profile of decoding accuracies (Table S6) and their exceedance probability (Figure 3D) are similar across the three decision strategies.

**Supporting References**

1. Werner-Reiss U, Groh JM (2008) A rate code for sound azimuth in monkey auditory cortex: implications for human neuroimaging studies. J Neurosci 28: 3747-3758.

2. Bonath B, Noesselt T, Martinez A, Mishra J, Schwiecker K, et al. (2007) Neural basis of the ventriloquist illusion. Curr Biol 17: 1697-1703.

3. Bonath B, Noesselt T, Krauel K, Tyll S, Tempelmann C, et al. (2014) Audio-visual synchrony modulates the ventriloquist illusion and its neural/spatial representation in the auditory cortex. Neuroimage.

4. Bischoff M, Walter B, Blecker C, Morgen K, Vaitl D, et al. (2007) Utilizing the ventriloquism-effect to investigate audio-visual binding. Neuropsychologia 45: 578-586.

5. Stein BE, Meredith MA (1993) The merging of the senses. Cambridge, MA: The MIT Press.

6. Noppeney U, Ostwald D, Werner S (2010) Perceptual decisions formed by accumulation of audiovisual evidence in prefrontal cortex. The Journal of Neuroscience 30: 7434-7446.

7. Wozny DR, Beierholm UR, Shams L (2010) Probability matching as a computational strategy used in perception. PLoS Comput Biol 6.

8. Gaissmaier W, Schooler LJ (2008) The smart potential behind probability matching. Cognition 109: 416-422.
